# An Image-Based Mobile Health App for Postdrainage Monitoring: Usability Study

**DOI:** 10.2196/17686

**Published:** 2020-08-28

**Authors:** Chien-Hung Liao, Yu-Tung Wu, Chi-Tung Cheng, Chun-Hsiang Ooyang, Shih-Ching Kang, Chih-Yuan Fu, Yu-Pao Hsu, Chi-Hsun Hsieh, Chih-Chi Chen

**Affiliations:** 1 Department of Trauma and Emergency Surgery Linkou Chang Gung Memorial Hospital Chang Gang University Taoyaun Taiwan; 2 Department of Physical Medicine and Rehabilitation Linkou Chang Gung Memorial Hospital Chang Gang University Taoyaun Taiwan

**Keywords:** telemedicine, smartphone, surgical drainage, postdrainage care, mHealth

## Abstract

**Background:**

The application of mobile health (mHealth) platforms to monitor recovery in the postdischarge period has increased in recent years. Despite widespread enthusiasm for mHealth, few studies have evaluated the usability and user experience of mHealth in patients with surgical drainage.

**Objective:**

Our objectives were to (1) develop an image-based smartphone app, SurgCare, for postdrainage monitoring and (2) determine the feasibility and clinical value of the use of SurgCare by patients with drainage.

**Methods:**

We enrolled 80 patients with biliary or peritoneal drainage in this study. A total of 50 patients were assigned to the SurgCare group, who recorded drainage monitoring data with the smartphone app; and 30 patients who manually recorded the data were assigned to the conventional group. The patients continued to record data until drain removal. The primary aim was to validate feasibility for the user, which was defined as the proportion of patients using each element of the system. Moreover, the secondary aim was to evaluate the association of compliance with SurgCare and the occurrence of unexpected events.

**Results:**

The average submission duration was 14.98 days, and the overall daily submission rate was 84.2%. The average system usability scale was 83.7 (SD 3.5). This system met the definition of “definitely feasible” in 34 patients, “possibly feasible” in 10 patients, and “not feasible” in 3 patients. We found that the occurrence rates of complications in the SurgCare group and the conventional group were 6% and 26%, respectively, with statistically significant differences *P*=.03. The rate of unexpected hospital return was lower in the SurgCare group (6%) than in the conventional groups (26%) (*P*=.03).

**Conclusions:**

Patients can learn to use a smartphone app for postdischarge drainage monitoring with high levels of user satisfaction. We also identified a high degree of compliance with app-based drainage-recording design features, which is an aspect of mHealth that can improve surgical care.

## Introduction

Surgical drainage is a therapeutic procedure with multiple purposes, including relieving symptoms, bypassing occlusions, and monitoring postoperative conditions [1-4]. In patients with acute biliary diseases, such as acute cholecystitis or cholangitis, drainage can relieve symptoms and stabilize the patient’s condition [5-7]. Drainage can also postpone emergency surgery, undergoing interval surgery instead [8,9]. Sometimes, patients need to monitor their own drains because of a prolonged therapeutic course. Traditional postdischarge drainage care depends on medical professionals asking patients to record the amount and characteristics of the fluid drained. However, these self-report measures are not only unreliable in elderly adults and those with impaired cognition [10,11], but also time-consuming with regard to processing the data [12]. Inadequate monitoring and care might prolong drainage insertion, delay recovery, reduce quality of life, and induce sequential complications such as an electrolyte imbalance, dehydration, sepsis, or physical injury related to disruption of the drain placement [13,14]. Therefore, proper monitoring of the drainage status is a critical issue.

In the last decade, apps for mobile devices have radically changed modern lifestyles. Additionally, the healthcare sector has been enriched by numerous apps [15,16]. Because of the increase in popularity of this new technology, the World Health Organization (WHO) has defined these tools as electronic health (eHealth) and mobile health (mHealth) applications. As the ownership of mobile devices has become more common [17], patients and their caregivers are increasingly willing to use technology to access health care [18,19]. Several studies have demonstrated that mHealth technology improves the control of cardiac function and glycemic hemostasis, enhances medication compliance, and shortens hospital stays [20-24]. Additionally, prior research on app protocols for surgical patients has focused on routine procedures that already have a low baseline rate of postoperative and discharge complications [25-27]. Although the experience with using mHealth apps in surgical care is limited, it has been suggested that surgical patients benefit from this new technological mode of support [28,29].

We developed an internet-based remote app to monitor drainage and conducted a study to investigate the adequacy of the remote app with regard to helping patients and caregivers properly manage drains at home. This study focused on the feasibility and clinical value of the remote app for patients with percutaneous or surgical drainage.

## Methods

### Study Population

Patients who were eligible to participate in this study were adult inpatients (aged ≥20 years) in the acute care surgery department of a medical center. We enrolled patients who were undergoing percutaneous or surgical drainage of the biliary tract or peritoneal cavity at our department from May 2019 to October 2019. All patients who fulfilled the inclusion criteria were approached to participate in this study. Notably, patients were excluded if they had neurologic or cognitive disorders prohibiting their usage of the app or ability to give informed consent. To calculate the sample size, we used the following parameters: α=.05, a power of 80%, an enrollment ratio of 1.6, and a complication decreasing rate of 18%. We recruited 50 patients to participate in the app group and 30 patients to participate in the conventional group.

The institutional review board of Chang Gung Memorial Hospital approved this study protocol (201900495B0). A research assistant helped the enrolled patients and caregivers install the app on their smartphones and instructed them how to use the app before the operation. Before patients were enrolled in this study, the research assistant evaluated their familiarity with wearable devices and smartphones. If the patients were not confident about using these devices, we provided further instructions to their caregivers.

### SurgCare App

**Figure 1:**
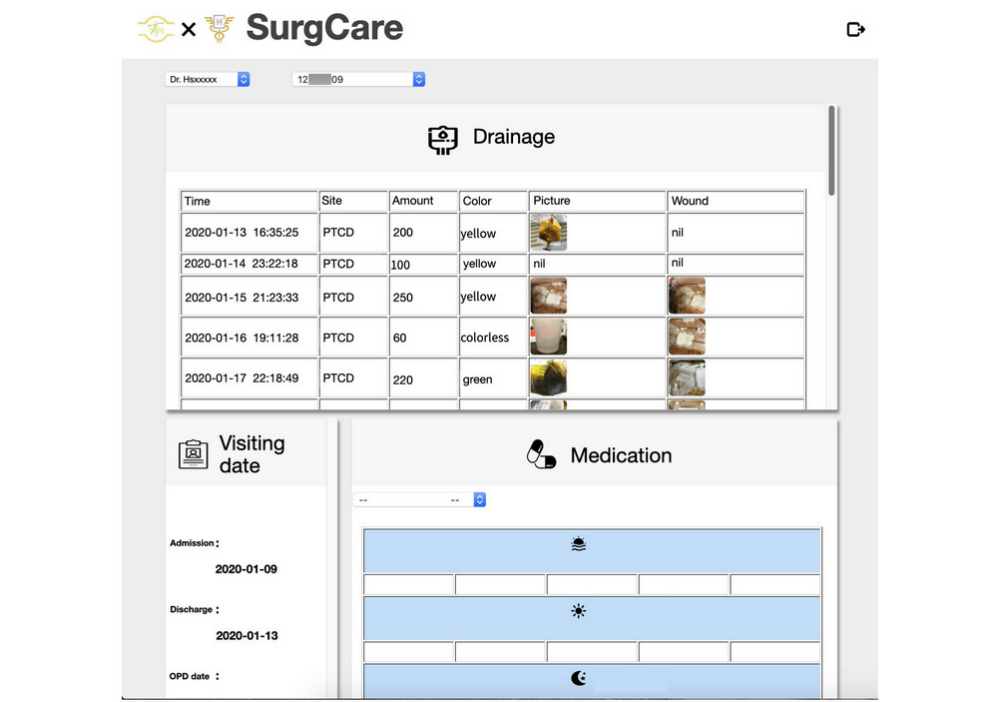
Screenshots of the SurgCare app showing the records of date, drain method, drainage amount, color, and image of drained material and wound status.

SurgCare (Figure 1) is an iOS/Android app that facilitates the recording of postprocedural clinical variables (drainage amount, discharge color, associated discomfort, body weight, and analgesic ingestion) by patients who have had drains placed.

**Figure 2:**
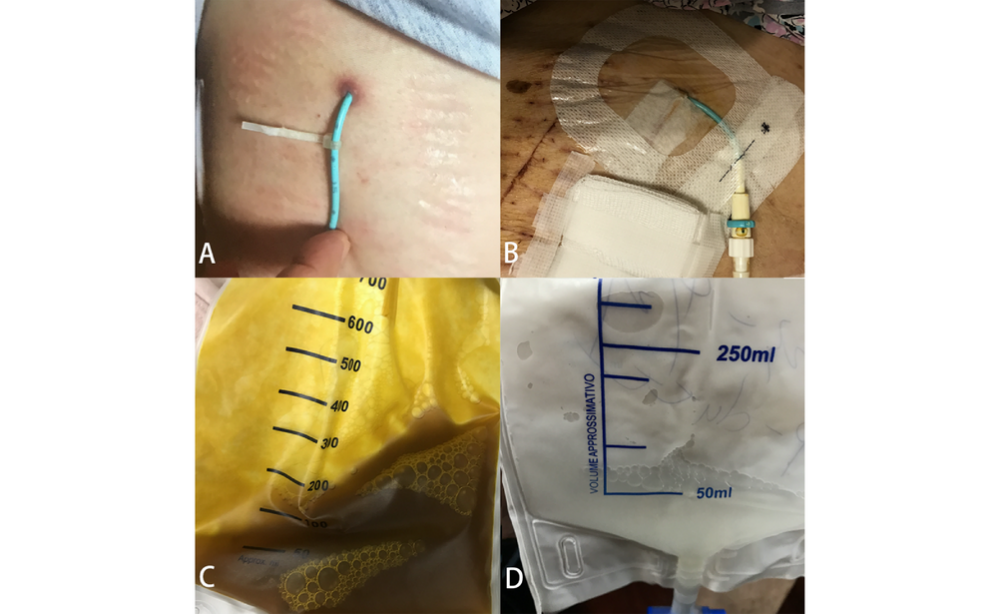
Image of drainage-inserted wound and drainage content. A: wound with percutaneous gallbladder drainage; B: wound with percutaneous biliary drainage; C: yellowish bile drainage; D: mucus-like white bile drainage.

The app transmits digital images of the surgical wound and drainage to the medical staff as shown in Figure 2.

**Figure 3:**
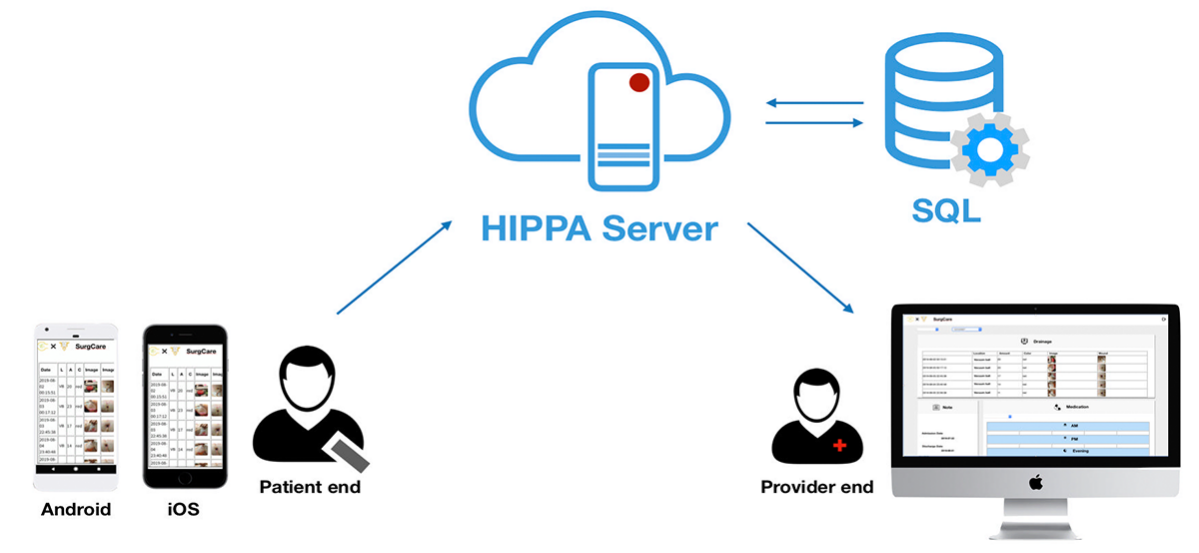
System architecture of the mobile device for recording postdrainage care.

SurgCare was developed by surgical professionals and software programmers to fulfill the needs of patients caring for drains at home and was only used by patients who were followed in our institute. Figure 3 presents the system architecture. Once the user inputs the data into the app, the information is synchronized with the server when internet access is available on the smartphone. This interdevice data transmission worked well, with no abnormal events reported by the patients.

Furthermore, the research assistant monitored the synchronizing of the data on weekdays and called the patients if their information was missing. If there was a change in fluid color, persistent changes in vital signs or abnormal drainage, this information was provided by smart devices to the medical doctors who then arranged further management. Patients continued to transmit data until their drains were removed. The research assistant did not have contact with the conventional group when patients were at home. Another medical staff member who did not participate in designing this system independently reviewed and analyzed the data.

### User Tasks

We formally tested the usability and feasibility of the app with postdischarge drainage patients at a major academic medical center. The app was loaded onto an iOS or Android smartphone or tablet. We assessed patients’ baseline familiarity with smartphones prior to testing. User tasks included waking up the device, launching the app, inputting information (including drainage amount, color, and the presence of discomfort), capturing an image, reviewing and retaking or accepting captured images, responding to questions, and submitting the data.

### Measures and Analysis

#### Feasibility: Protocol Completion

Following usability testing of the app, participants were asked to rate their performance and to provide feedback on the app. Participants also used a system usability scale to evaluate their satisfaction with the app [30].

We evaluated the compliance of the patients with the use of SurgCare. If patients submitted data on more than 80% of days on which they had a drain, they were classified in the good compliance group. If the number of days on which data were submitted was less than 80% of the total number of days for which they had the drains, we classified them in the poor compliance group.

In this study, the feasibility was assessed as in past studies [31]. The feasibility was defined as the proportion of participants using each element of the system for at least 70% of the period. “Definitely feasible,” “possibly feasible,” and “not feasible” were defined as ≥70%, 50%-69%, and <50% of the participants meeting that criterion, respectively.

#### Clinical Value: Association of App Usage With Early Complication Rate

The clinical outcome of interest was validation that the use of the app leads to fewer complications and a lower incidence of unexpected hospital return. We compared the high compliance group with the poor compliance group and conventional group with regard to the total compliance rate, incidence of drain dysfunction, incidence of drain dislodgement, and rate of infection. We also analyzed the rates of unexpected hospital return and readmission in these groups.

### Statistical Analysis

Pearson’s chi-square test and Fisher exact test were used to compare categorical variables. Quantitative variables were compared with Student’s *t* test. Levene’s test was used to correct for intergroup variations before the application of Student’s *t* test. Statistical analysis was performed with SPSS v 20.0 for Macintosh (SPSS Inc). A value of *P*<.05 was considered statistically significant.

## Results

### Participant Characteristics

**Figure 4:**
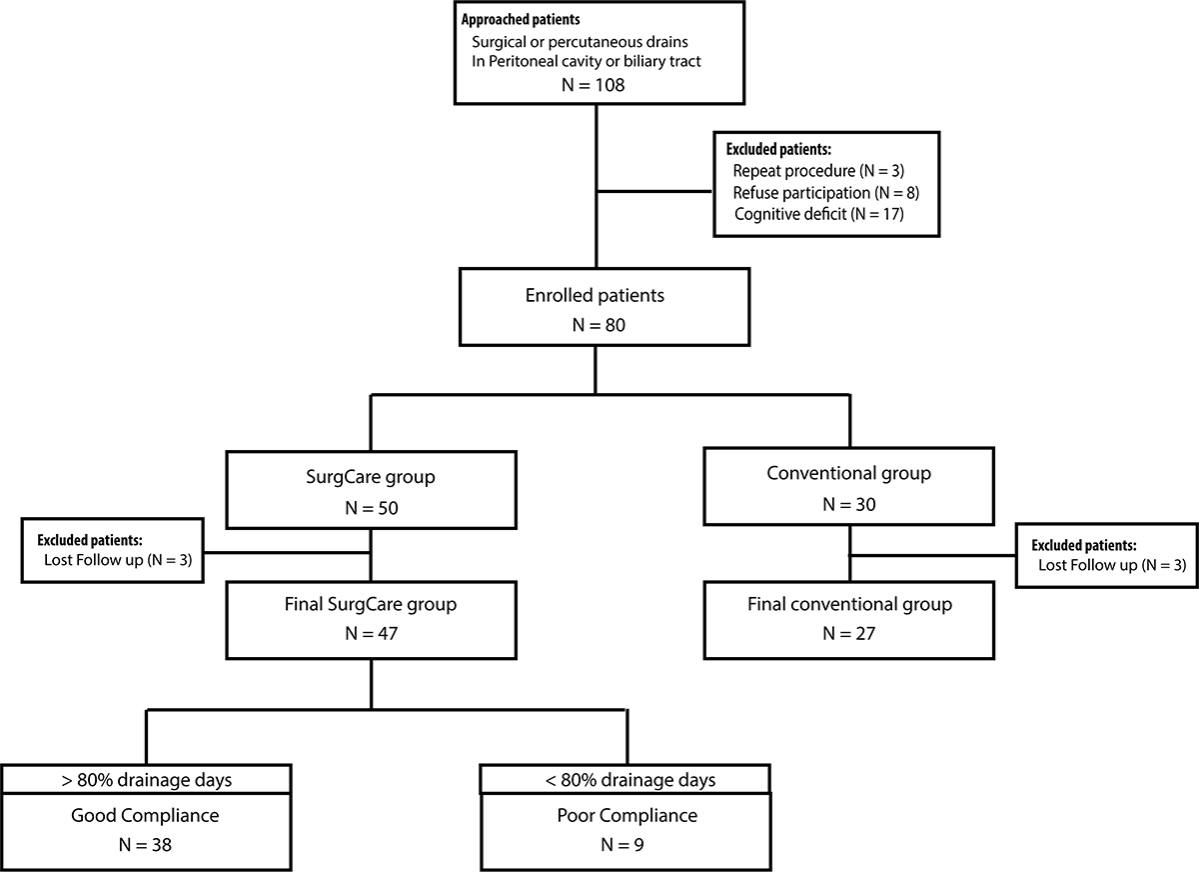
Flow diagram of the study design.

In total, 105 patients underwent 108 procedures during the study period. Out of these 105 patients, 3 patients with repeated procedures were excluded. After being approached, 8 patients refused to participate in this project, and another 14 patients had cognitive problems and were excluded from this study. Of the 80 patients who were approached, were eligible, and agreed to participate, 6 patients were lost to follow-up. A total of 47 participants completed the usability testing, 26 of whom had caregiver assistance or proxy participation. Another 27 patients were included in the conventional group; these patients used the traditional hand recording method to track the drainage amount and color. The study flow diagram is shown in Figure 4.

Demographics and basic clinical information of SurgCare group are presented in Table 1. The average duration of app use was 14.5 (SD 3.2) days. The common termination of follow-up was drainage removal and wound closure, followed by a lack of satisfaction and complications.

**Table 1:** Demographic characteristics of SurgCare participants with surgical and percutaneous drainage (N=47).

Characteristics		Value
Age, mean (SD)		61.4 (15.9)
**Gender, n (%)**		
	Male	29 (62)
	Female	18 (38)
**Underlying disease, n (%)**		
	Hypertension	21 (45)
	Diabetes mellitus	16 (34)
	Renal insufficiency	7 (15)
	Chronic obstructive pulmonary disease	3 (6)
	Malignancy	3 (6)
**Drainage site, n (%)**		
	Biliary drainage	25 (53)
	Peritoneal drainage	22 (47)
**Method of participation, n (%)**		
	Independent	21 (45)
	Caregivers	26 (55)
System usability scale, mean (SD)		83.7 (3.5)
**Feasibility test, n (%)**		
	Definite feasible	34 (72)
	Possibly feasible	10 (21)
	Not feasible	3 (6)
**Overall daily compliance**		
	Total drainage days, N	836
	Day submitted, n (%)	704 (84)
	Day missed, n (%)	132 (16)
**Complication, n (%)**		
	Dysfunction	1 (2)
	Dislodge	0 (0)
	Infection	2 (4)
	Unexpected return	3 (6)
	Unexpected readmission	2 (4)

To compare SurgCare group with the conventional group, we identified gender, age, underlying chronic disorders, drainage site, and method of participation for both the groups. Total occurrence of complication was lower in SurgCare group (6%) than in the conventional group (26%) with statistical significance of *P*=.03. The incidence of drainage dislodge was lower in SurgCare group (2%) than in the conventional group (11%) with statistical significance of *P*=.045. Moreover, the unexpected hospital return was lower in SurgCare group (6%) than in the conventional group (26%) with significant difference of *P*=.03 (Table 2).

**Table 2:** Comparison of the characteristics and prognosis of patients within the SurgCare and conventional groups.

Characteristics		SurgCare	Conventional	*P* value
Patients, n		47	27	—^a^
Age, mean (SD)		60.2 (17.1)	63.6 (13.4)	.36
**Gender, n (%)**				.60
	Male	29 (62)	15 (56)	
	Female	16 (38)	12 (44)	
**Underlying disease, n (%)**				
	Hypertension	21 (45)	8 (30)	.23
	Diabetes mellitus	16 (34)	6 (22)	.43
	Renal insufficiency	7 (15)	5 (19)	.75
	Chronic obstructive pulmonary disease	3 (6)	2 (7)	>.99
	Malignancy	3 (6)	3 (11)	.66
**Drainage site, n (%)**				.08
	Biliary drainage	25 (53)	20 (74)	
	Peritoneal drainage	22 (47)	7 (26)	
**Method of participation, n (%)**				.37
	Independent	21 (45)	15 (56)	
	Caregivers	26 (55)	12 (44)	
**Complication, n (%)**		3 (6)	7 (26)	.03^b^
	Dysfunction	1 (2)	3 (11)	.14
	Dislodge	0 (0)	3 (11)	.045^b^
	Infection	2 (4)	1 (4)	>.99
	Unexpected hospital return	3 (6)	7 (26)	.03^b^
	Unexpected readmission	2 (4)	4 (15)	.11

^a^Not applicable.

^b^Fisher Exact test.

### Feasibility and Usability Evaluation of SurgCare

After evaluation, there were 34 patients for whom it was definitely feasible to use this app. For 10 patients, use of the app might be feasible, although they needed more support from the research assistant to help them operate the system. Another 3 patients were in the infeasible group because they completed less than 30% of the elements. The overall system usability score for the app was 83.3, which is considered good in usability testing.

### Association of SurgCare App Usage Compliance and Early Complication Rate

In the good compliance group, we found that the rate of complications related to drainage was 3%, which was much lower than that in the poor compliance group (11%). With regard to unexpected hospital return (3% vs 11%) and readmission (3% vs 11%), the good compliance group had better results than the poor compliance group and the conventional group as shown in Table 3.

**Table 3:** Comparison of the prognosis of patients with SurgCare with good and poor compliance.

Characteristics		Good compliance	Poor compliance	*P* value	
Patients, n		38	9	—^a^	
Age, mean (SD)		60.3 (18.1)	60.0 (13.1)	.96	
**Gender, n (%)**					.27
	Male	22 (58)	7 (78)		
	Female	16 (42)	2 (22)		
**Underlying disease, n (%)**					
	Hypertension	16 (42)	5 (56)	.47	
	Diabetes mellitus	15 (40)	1 (11)	.11	
	Renal insufficiency	7 (18)	0 (0)	.32	
	Chronic obstructive pulmonary disease	1 (3)	2 (22)	.09	
	Malignancy	2 (5)	1 (11)	.48	
**Drainage site, n (%)**					.87
	Biliary drainage	20 (53)	5 (56)		
	Peritoneal drainage	18 (47)	4 (44)		
**Method of participation, n (%)**					.14
	Independent	15 (40)	6 (67)		
	Caregivers	23 (61)	3 (33)		
**Complication, n (%)**					
	Dysfunction	1 (3)	0 (0)	>.99	
	Dislodge	0 (0)	0 (0)	>.99	
	Infection	1 (3)	1 (11)	.32	
	Unexpected hospital return	2 (5)	1 (11)	.52	
	Unexpected readmission	1 (3)	1 (11)	.26	

^a^Not applicable.

## Discussion

This study demonstrated the feasibility of using a mobile app to monitor the recovery status of patients with drains and to assist patients and caregivers in detecting abnormalities in a timely manner. Remote apps could support self-care and allow close follow-up [32-34]. We also identified an unexpected reduction in the rates of hospital return and readmission in the SurgCare group (6.4%) compared with the conventional group (25.9%) (*P*=.02). We found that the occurrences of complications such as dislodgement (0%), infection (4%), and dysfunction (2%) were relatively fewer in the SurgCare group. To the best of our knowledge, this is one of the first innovative studies focusing on the development of comprehensive app functions to assist surgical patients with drain care and monitoring. With the evaluation of uploaded image of wound and drainage content, the health care team can identify the abnormalities earlier to prevent the sequential complications, which is one of the causes to reduce unexpected hospital return. The current standard of care for the majority of surgical patients following hospital discharge involves little formal communication between patients and their care team until their routine clinic follow-up 2-3 weeks after discharge [35,36]. Some mHealth protocols have been developed to improve patient monitoring or replace routine postoperative clinic visits [37-39]. In addition to this trend, the national policy priority mandates improving transitions of care following hospital discharge and reducing hospital readmissions [40,41]. We created an image-based mobile app aimed at increasing communication between patients and health care personnel after discharge from the hospital as part of an effort to detect drainage complications in an early stage and reduce hospital readmissions.

The compliance with using apps is generally high. Apps for surgical patients must be developed carefully, keeping in mind that the users are very vulnerable [42,43]. In this study, we find that compliance with using the SurgCare was acceptable. Most participants found the app easy to use (%), though the questions that did not elicit an unanimously positive response indicate that there is a degree of tentativeness regarding participants’ ability to independently perform the functions in the SurgCare. The SurgCare can provide assistance not only by facilitating monitoring but also by providing psychological support [44,45]. For patients and caregivers who are not familiar with postoperative wound care, remote support helps them detect the early dislodgement or dysfunction of the drain, and it can reduce the subsequent occurrence of infections and other complications. Self-report questionnaires are the most common method of monitoring drainage because of their cost-effectiveness and ease of administration [46]. However, the disadvantages of self-report questionnaires include a lack of reliability and the influences of social desirability, age, questionnaire complexity, and recall ability [47,48]. Therefore, an easily used mobile app can solve these problems. With immediate recording and image capture, the app is an excellent tool for close monitoring. Moreover, SurgCare can offer a rapid response to help patients psychologically, as patients and caregivers can receive responses from healthcare personnel before returning to the clinic. The daily monitoring messages are delivered via mobile messaging (eg, short message service) instead of email to facilitate more immediate communication. These adjustments are expected to improve user satisfaction and compliance and may ultimately further enhance the efficacy of the intervention.

Furthermore, 4 key categories of age-related barriers are associated with the use of mHealth by older adults, namely, barriers related to cognition, motivation, physical ability, and perception [26]. As surgical drainage patients are usually older adults (median age >60 years) who have the potential to develop cognitive or memory impairments, it is crucial to select an easy-to-follow app to use in clinical research with this population. We noticed that if the patients were well trained, they were able to input their health data by themselves without any dependence on a physician’s assistant, study nurse, or other caregivers [49,50]. After the interview, we also noticed that the surgeons were interested in the electronic assessment of patient-reported outcomes rather than the conventional manual reporting of the drainage amount and status. Previous studies have suggested that the electronic assessment of patient-reported outcomes was as accurate as the conventional method of manual recording [51,52]. Another survey of 108 health care personnel showed a high level of acceptance (84.3%) of app-assisted recording [53]. Digital medicine is unstoppable, and patient empowerment plays a new and growing role in disease management. With support from both patients and health providers, we can determine the impact of mHealth on the postprocedural care of surgical drains.

In contrast to the generally rapid growth of mHealth in medical fields, research on surgical topics has been limited. Among studies focusing on the application of mHealth to surgical issues, several have investigated wound care and pain scaling to validate the clinical usefulness of mHealth [54-57]. A recent study used another wearable device to track the step counts of patients who had undergone various abdominal surgeries for 1 month after discharge and showed that the mHealth app could effectively track recovery [24,58]. Because drain care is an issue that is unique to surgical patients, and telephone conversations and questionnaires cannot be used to access the visual component. In this study, we developed an app that can improve compliance with postdischarge drainage care and monitoring and reduce the risk of drainage complications.

### Limitations

This study had several limitations that should be considered when interpreting the results. First, all assessments were conducted online, and inclusion relied exclusively on self-reported data. Therefore, the internal validity and generalizability to a larger clinical population might have been compromised. Second, there was a rather low postassessment response rate. The lack of data from approximately one-fifth of the sample limits the validity of our findings since it remains unclear how satisfied the nonresponders were and how they differed in terms of symptomatology. Third, the sample size was limited, and the sample did not represent all surgical patients. Subsequent research should, therefore, investigate the efficacy and cost-effectiveness of SurgCare in a fully powered randomized control trial. The study provided valuable information about the feasibility and adequacy of an internet-based intervention for the management of drains, which can be used to guide subsequent research.

### Conclusion

In this study, we present a remote app that can improve patient compliance with postdischarge drainage care and monitoring and reduce the rate of major complications. The patients were enthusiastic about partnering with their health providers in novel ways to optimize their healthcare. Although mHealth will certainly not replace physician contact, it will serve as a digital assistant for diagnostic, therapeutic, and follow-up purposes, supporting patient recovery.

## Abbreviations

eHealth: electronic health
mHealth: mobile health
WHO: World Health Organization
